# Genomic Diversity of *Mycobacterium tuberculosis* Complex Strains in Cantabria (Spain), a Moderate TB Incidence Setting

**DOI:** 10.1371/journal.pone.0157266

**Published:** 2016-06-17

**Authors:** Inmaculada C. Pérez del Molino Bernal, Troels Lillebaek, Mathias K. Pedersen, Luis Martinez-Martinez, Dorte B. Folkvardsen, Jesús Agüero, E. Michael Rasmussen

**Affiliations:** 1 Service of Microbiology, University Hospital Marqués de Valdecilla, Santander, Spain; 2 International Reference Laboratory of Mycobacteriology, Statens Serum Institut, Copenhagen, Denmark; 3 Valdecilla Biomedical Research Institute (IDIVAL), Santander, Spain; 4 Department of Molecular Biology, School of Medicine, University of Cantabria, Santander, Spain; Chang-Gung University, TAIWAN

## Abstract

**Background:**

Tuberculosis (TB) control strategies are focused mainly on prevention, early diagnosis, compliance to treatment and contact tracing. The objectives of this study were to explore the frequency and risk factors of recent transmission of clinical isolates of *Mycobacterium tuberculosis* complex (MTBC) in Cantabria in Northern Spain from 2012 through 2013 and to analyze their clonal complexity for better understanding of the transmission dynamics in a moderate TB incidence setting.

**Methods:**

DNA from 85 out of 87 isolates from bacteriologically confirmed cases of MTBC infection were extracted directly from frozen stocks and genotyped using the mycobacterial interspersed repetitive units-variable number tandem repeat (MIRU-VNTR) method. The MIRU-VNTR*plus* database tool was used to identify clusters and lineages and to build a neighbor joining (NJ) phylogenetic tree. In addition, data were compared to the SITVIT2 database at the Pasteur Institute of Guadeloupe.

**Results:**

The rate of recent transmission was calculated to 24%. Clustering was associated with being Spanish-born. A high prevalence of isolates of the Euro-American lineage was found. In addition, MIRU-VNTR profiles of the studied isolates corresponded to previously found MIRU-VNTR types in other countries, including Spain, Belgium, Great Britain, USA, Croatia, South Africa and The Netherlands. Six of the strains analyzed represented clonal variants.

**Conclusion:**

Transmission of MTBC is well controlled in Cantabria. The majority of TB patients were born in Spain. The population structure of MTBC in Cantabria has a low diversity of major clonal lineages with the Euro-American lineage predominating.

## Introduction

The incidence of TB has gradually decreased worldwide, but remains a major global health problem with 1.5 million deaths in 2013 [1]. In Spain, the overall incidence was 11.8 TB cases per 100,000 inhabitants in 2013 [2]. Same year, in Cantabria, a coastal region located in the North of Spain, a similar incidence of 12.4 TB cases per 100,000 inhabitants was observed [2]. This is far from the eradication target stated by WHO for 2050 defined as less than one case per million inhabitants per year in developed countries [1].

During the last two decades, DNA genotyping techniques have been used to study MTBC strains, in particular issues related to recent transmission of the bacteria [3–13]. Generally, it is assumed, that isolates with identical DNA genotypes represent recent transmission, but other variables must be taken into account before concluding on recent transmission. For instance, the presence or absence of epidemiological links, the accuracy of the genotyping methods, the grade of strain diversity and the relation between cases in time and location influence the interpretation [8, 14, 15]. MIRU-VNTR [16] and whole genome sequencing (WGS) [12] have increased our knowledge and helped appreciate the clonal complexity of MTBC [17–20] as well as its phylogeographical distribution [21, 22] and the impact that several lineages have on host immune regulation, transmission and disease severity [23].

In the present study, 85 MTBC strains from the region of Cantabria from 2012 through 2013 were genotyped with the 24 locus-based MIRU-VNTR method to analyze the genomic diversity of MTBC and to estimate the frequency and risk factors associated with recent transmission.

## Materials and Methods

Cantabria is a coastal, mountainous region with 591,888 inhabitants [24], located in the North of Spain. The majority of the population lives in the coastal area. In 2012, only 6.5% of the population in Cantabria was immigrants compared to 11.8% in the rest of Spain [24]. Most people in the region, including immigrants and homeless, have access to the public health service without charge.

### Study population and design

This was a retrospective cohort study. The cohort included all patients living in Cantabria who presented with pulmonary and/or extrapulmonary TB confirmed by culture from January 1st 2012 to December 31st 2013. The population of analyzed MTBC (n = 85) represent 98% of bacteriologically verified cases (n = 87) and 61% (n = 139) of all TB cases reported in Cantabria in the period. The cases were enrolled from all five hospitals treating TB patients in the region of Cantabria. Epidemiological, clinical and microbiological data were gathered from the medical case records and the Department of Microbiology at University Hospital Marqués de Valdecilla. One isolate from each patient was included and genotyped.

### Laboratory methods

All the sputum samples were decontaminated and cultured on Coletsos slants (Difco, Maryland, USA) and BacT/ALERT-3D seed bottles (BioMérieux, Marcy l'Etoile, France). MTBC was identified by 16S ribosomal RNA hybridization technique (AccuProbe; Gen Probe Inc., San Diego, California). The cultures were verified and susceptibility tested using the method of critical concentrations at the National Microbiology Centre, Institute of Health Carlos III, Madrid, Spain. The isolates were suspended in 1.5 ml of storage medium (BHI + glycerin 20%) and kept at -80°C until DNA analysis. DNA extraction was performed directly from stock and MIRU-VNTR typing performed as described by Supply et al. [16]. For the 24 loci based MIRU-VNTR typing we used a commercial kit (Genoscreen, Lille, France) and processed with a 48-capillary ABI 3730 DNA Analyzer (Applied Biosystems, CA, USA). The MIRU-VNTR allele assignation was performed using GeneMapper software (Applied Biosystems, CA, USA) [16]. Genotyping was performed at the International Reference Laboratory of Mycobacteriology (IRLM) at Statens Serum Institut in Copenhagen, Denmark. In the external quality control for 24 locus MIRU-VNTR typing performed by the European Center for Disease Prevention and Control (ECDC) in 2014, IRLM obtained typing results matching 100% with the reference data.

### Data analysis

The international online MIRU-VNTR*plus* database tool was used to identify clusters, lineages and to build neighbor joining (NJ) phylogenetic tree (www.miru-vntrplus.org) [25, 26]. We used the strategy proposed by Allix-Béguec et al. for optimal phylogenetic identification [25]. When two or more MTBC isolates were 100% identical by MIRU-VNTR, the cases were classified as clustered [27]. Strains with more than one allele at a single locus were classified as clonal variants infection within a host, whereas strains with more than one allele at two or more loci were classified as simultaneous coinfection by more than one *M*. *tuberculosis* strain [16, 17, 28]. These cases were confirmed by repeating MIRU-VNTR with a new extraction of DNA from the stock.

The clustering rate was calculated as (n_c_-c)/n, where n_c_ was the total number of clustered cases, c was the number of clusters and n was the total number of genotyped isolates [3]. The epidemiological and clinical data of the patients were compared between the clustered and non-clustered cases in the study.

In order to know if the clusters were already described, we compared our MIRU-VNTR types with the MIRU-VNTR international types (MIT) of SITVIT2 database [29].

Chi-square test or Fisher’s exact test were performed to test the univariate risk factors for belonging to a cluster. The statistical analysis of data was performed using the software SPSS version 19 (IBM Corporation, NY, USA), excluding all cases with missing data. A p value <0.05 was considered significant. Statistical analysis was not performed when the expected cell frequencies were equal to zero.

### Ethics approval

This project was approved by the ethics committee of Cantabria (CEIC-2013.252).

## Results

### Patient characteristics

Forty-eight (56.5%) cases were males and 37 (43.5%) cases females. The median age was 50 years (range 4 to 89 years). The highest proportions of cases were found in the age groups 40–59 (47%) and 20–39 (27%). The majority of patients were Spanish-born (80%) in contrast to foreign-born (20%). The foreign-born were immigrants from South-Central America (71%), Europe (23%) and Africa (6%). Roughly an equal number of patients were living in urban and in rural areas. There were more pulmonary (82.4%) than extra-pulmonary cases (17.6%). Among the pulmonary cases, 77% had positive sputum smears and 50% of them had cavities on chest X-ray (CXR). Among sputum smear negative cases, only 13% had cavities on CXR. In total, 78 (91.8%) strains were susceptible to first and second line drugs, six (7%) were monoresistant (three to isoniazid; two to streptomycin and one to pyrazinamide) and one (1.2%) was multidrug resistant.

### MIRU-VNTR typing

A total of 85 isolates (98%) were successfully typed with MIRU-VNTR; 83 were identified as *M*. *tuberculosis*, one as *M*. *bovis* and one as *M*. *africanum*. Two cases were not typed due to insufficient DNA for analysis and no amplification signal in PCR, respectively.

The MIRU-VNTR analysis detected a total of 65 different patterns; 31 isolates (36%) were grouped in 11 clusters with different MIRU-VNTR types and 54 (64%) were unique patterns.

The clustering rate was 24%. The size of clusters varied between two and five patients, with only one cluster of five cases and five clusters with only two patients. The mean age of the patients in clusters was 50.5 years; 18 (58.1%) were men and 13 (41.9%) women. The most common clinical presentation was pulmonary TB (87.1%). Twenty-nine (93.5%) patients were born in Spain and two (6.5%) patients were foreign-born. The comparison of clustered and non-clustered patients is shown in Table 1. Statistical analysis of potential causes for clustering revealed that nationality was associated with clustering (p = 0.023)—a Spanish-born was more likely to be clustered than a foreign-born. No other variables were found to be significantly associated with clustering.

**Table 1:** Comparison of socio-demographic and clinical characteristics of clustered and non-clustered TB cases.

Parameters	Clustered	Non clustered	Total	*p value*
**N°**	**31(%)**	**54(%)**	**85**	
**Age group**				
**<35**	**5(16.1%)**	**12(22.2%)**	**17(20%)**	**0.499**
**>35**	**26(83.9%)**	**42(77.8%)**	**68(80%)**	
**Gender**				
**Male**	**18(58.1%)**	**30(55.6%)**	**48(56.5%**	**0.822**
**Female**	**13(41.9%)**	**24(44.4%)**	**37(43.5%)**	
**Inmigrant**				
**Spanish-born**	**29(93.5%)**	**39(72.2%)**	**68(80%)**	**0.023**
**Foreign-born**	**2(6.5%)**	**15(27.8%)**	**17(20%)**	
**Place of residence**^a^				
**Rural**	**15(48.4%)**	**25(46.3%)**	**40(47.1%)**	**0.853**
**Urban**	**16(51.6%)**	**29(53.7%)**	**45(52.9%)**	
**Site of TB**				
**Extrapulmonary**	**4(12.9%)**	**11(20.4%)**	**15(17.6%)**	**0.556**
**Pulmonary**	**27(87.1%)**	**43(79.6%)**	**70(82.4%)**	
**HIV status**				
**HIV-**	**31(100%)**	**43(79.6%)**	**74(87.1%)**	
**HIV+**	**0**	**5(9.3%)**	**5(5.9%)**	
**ND**	**0**	**6(11.1%)**	**6((7%)**	
**Alcohol use**				
**Non**	**16(51.6%)**	**36(66.7%)**	**52(61.2%)**	**0.255**
**Yes**	**11(35.5%)**	**14(25.9%)**	**25(29.4%)**	
**ND**	**4(12.9%)**	**4(7.4%)**	**8(9.4%)**	
**Smoking**				
**Non**	**19(61.3%)**	**27(50%)**	**46(54.1%)**	**0.204**
**Yes**	**10(32.2%)**	**26(48%)**	**36(42.4%)**	
**ND**	**2(6.5%)**	**1(2%)**	**3(3.5%)**	
**Smear**				
**Positive**	**21(67.7%)**	**39(72.3%)**	**60(70.6%)**	**0.475**
**Negative**	**10(32.3%)**	**13(24%)**	**23(27.0%)**	
**ND**	**0**	**2(3.7%)**	**2(2.3%)**	
**Chronic disease**^b^				
**Non**	**13(42%)**	**29(53.8%)**	**42(49.4%)**	**0.058**
**Yes**	**15(48.3%)**	**13(24%9**	**28(33%)**	
**ND**	**3(9.7%)**	**12(22.2%)**	**15(17.6%)**	

^**a**^ Rural <30.000 inhabitants Urban > = 30.000 inhabitants

^**b**^ Diabetes, malignant disease, immunosuppression not mediated by HIV

**ND**: Not data available.

**Fig 1:**
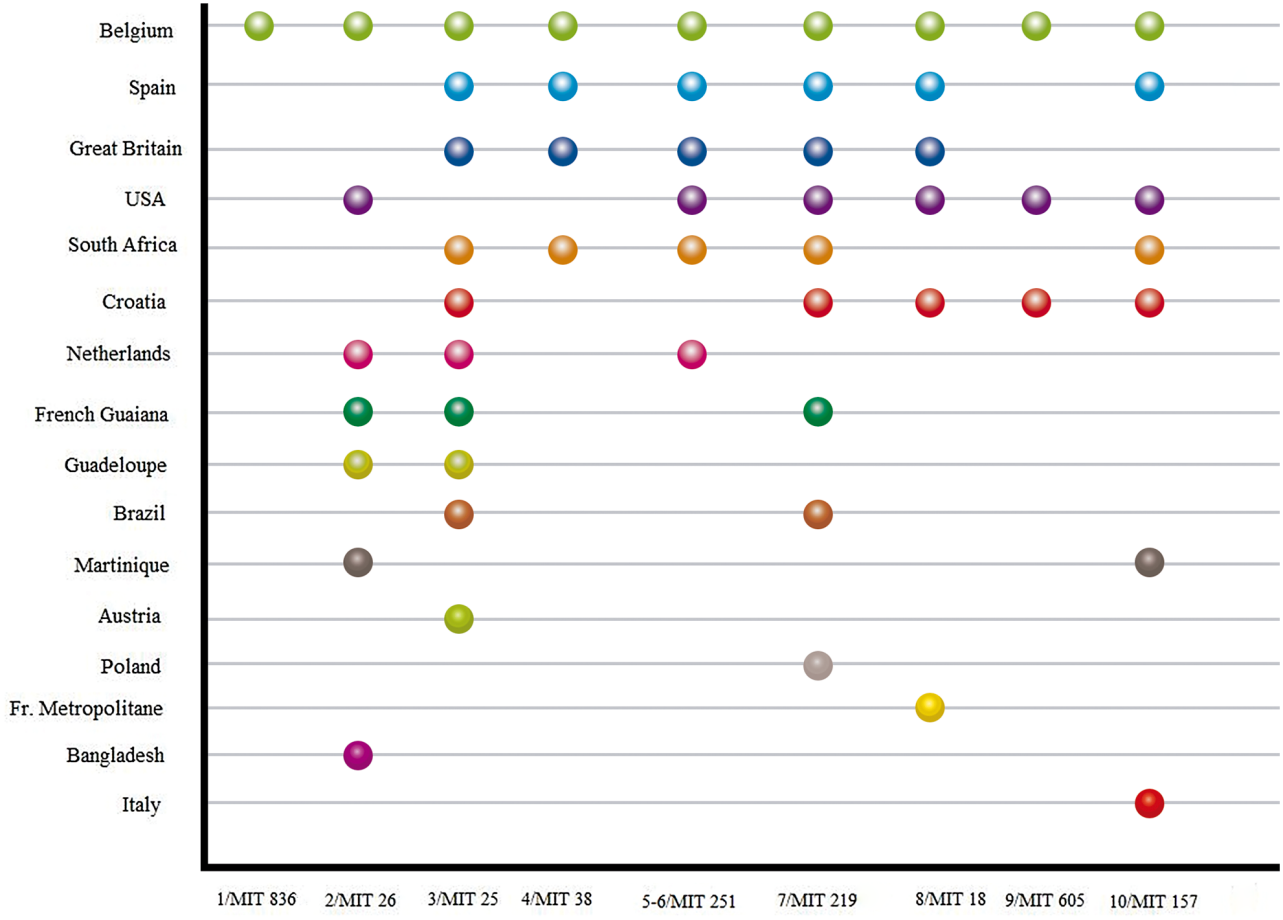
Cluster comparison. Comparison of identical (or one allele different = 1/MIT 836 and 7/MIT0219) clusters in Cantabria and MIRU-VNTR International Types (MIT, reported in reference 29 doi: 10.1016/j.meegid.2012.02.004).The comparison is based on MIRU12.

In ten of the clustered MIT profiles, we identified the MIT either by exact match or with only one allele difference (Fig 1). The profiles were clustering mainly with isolates from Belgium, Great Britain, USA, Croatia, South Africa and also Spain. The cluster number 10, MIT157, was shared with the *M*. *tuberculosis Zaragoza* (*MTZ*) strain described in 2009 (30) and the clusters 4 and 8 (MIT38 and MIT18) were shared with Madrid1 and Madrid 2 (31) clusters respectively.

Of the 85 typed strains, six (7%) strains presented a double allele in a single MIRU-VNTR locus. The corresponding loci were 2163b (QUB-11b) (two strains), 4052 (QUB-26), 3192 (MIRU 31), 3690 (Mtub-39) and 4156 (QUB-4156). Among these patients, two were immigrants from Romania and Republic of Moldova respectively, and among the Spanish-born patients, two had previously had TB. All these strains were susceptible to first line drugs. All these patients had pulmonary TB, five were unilateral and one compromised both lungs.

### Phylogenetic lineages

The Euro-American lineage was the most widespread (98%), including 49 strains (58%) of five known sublineages, and 34 strains (40%) with no identified (NI) sublineages, using the MIRU-VNTR*plus* database. One West African I (1%) and one *M*. *bovis* (1%) complete the picture of lineages in Cantabria.

**Fig 2:**
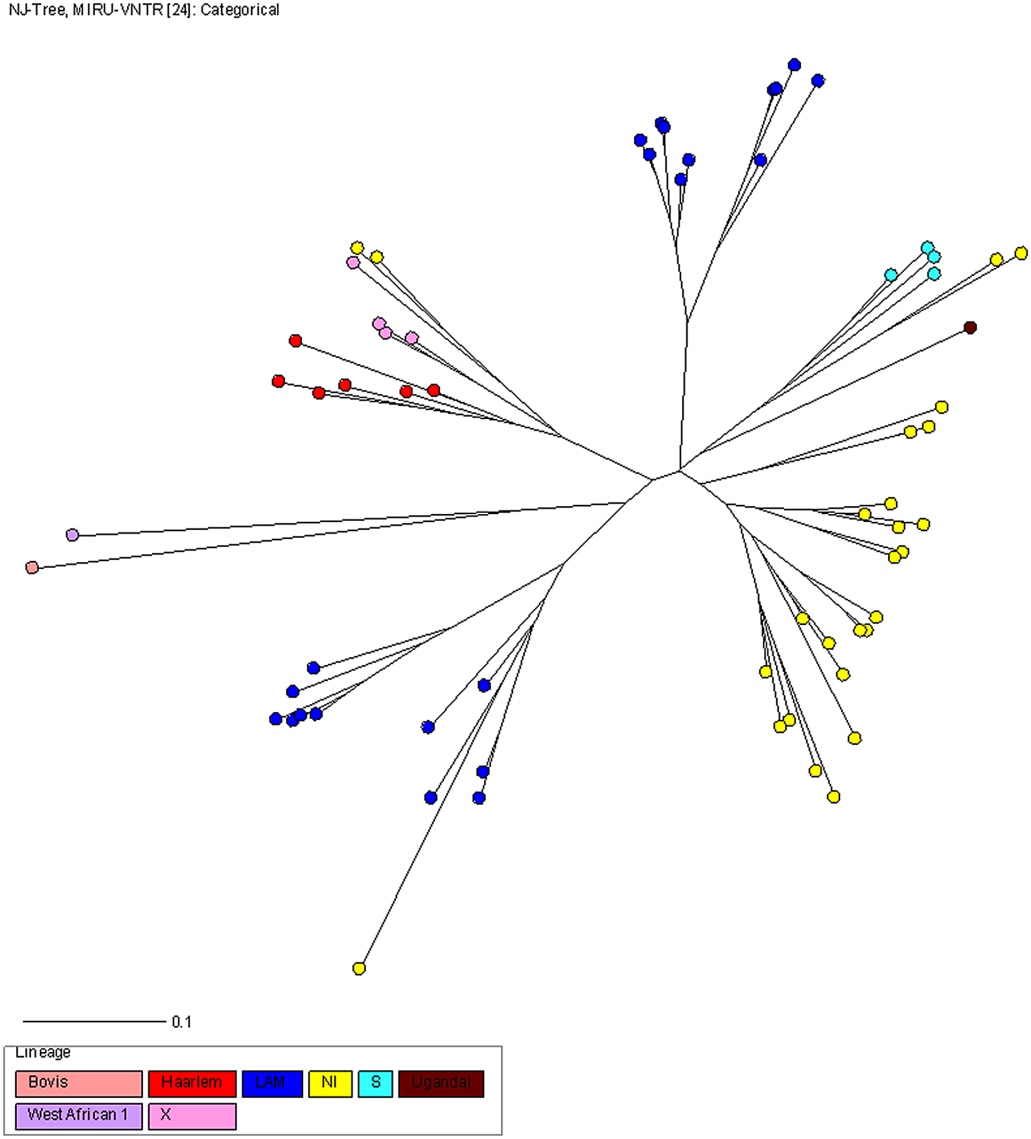
NJ phylogeny tree rooted by Canetti based on 24-loci MIRU-VNTR data. Colors indicate the main MTBC lineages. Each circle represents a MIRU-VNTR genotype and the circle size is proportional to the number of strains in cluster.

Latin American Mediterranean (LAM), n = 34 (40%), and NI, n = 34 (40%), were the predominant sublineages. Furthermore, four other Euro American sublineages (18%) (S, Haarlem, Uganda -I, X) were observed. As can be seen in Fig 2, MIRU-VNTR typing showed highly linked strains belonging to three main branches, two for LAM sublineages and one for NI sublineages. Only LAM and NI sublineages were found in clusters as defined by MIRU-VNTR type.

## Discussion

We conducted a population-based study to analyze the genomic diversity of MTBC and to determine the frequency of recent transmission as well as associated risk factors in the region of Cantabria, Spain, an area of moderate TB-incidence and a low rate of immigration. We demonstrated an MTBC population with a low clustering rate (24%) implying limited ongoing transmission within the region. Clustering was associated with being Spanish-born. A high prevalence of Euro-American lineage was found. Ten of our MIT clustered patterns have been found in other countries such as Belgium, Great Britain, USA, Croatia, South Africa and The Netherlands and six (7.1%) of the strains analyzed presented clonal variants.

Of the cases included in the study, 17 (20%) were isolates from the foreign-born population. This is similar to observations made in Zaragoza, Spain (17.6%) [10], but lower than observed in Almeria, Spain (52.6%), which has a higher immigration rate than Cantabria [11]. Our region had experienced an increasing immigrant rate until 2000, followed by a downward trend in the following years [24]. This fact, could be one of the reasons for the lower number of foreign-born cases found in this group. Furthermore, the disappearance of the health card for patients without residence papers since 2012 could lead us to underestimate the size of foreign population with TB.

In high-income countries and cities, the proportion of new cases of TB due to recent transmission versus reactivation is higher [3, 8, 10, 11]. We found a clustering rate of 24% that is lower than Denmark (47%) [4], San Francisco (31%) [3] and other cities in Spain (Gran Canaria (58.5%) [8], Zaragoza (42.7%) [10], Almeria (32%) [11]) but higher than Zurich (11%) [5], a place with lower ongoing transmission. Different circumstances could explain our low rate. Firstly, as 39% of all TB cases could not be bacteriological verified and therefore genotyped, clustering could be underestimated [15]. Secondly, the majority of residents have free and universal access to public health services with a mycobacteriology lab open every day of the week to evaluate cases with high suspicion of active TB. Finally, reporting of all cases of active TB to the Regional Health Service is mandatory for the physician involved in the diagnosis. As these factors contribute to faster TB control, the clustering rate would tend to be lower.

Several risk factors have been associated with clustering: age younger than 60 years, male sex, positive smear microscopy, previous contact with a TB patient, injection drug use, urban residence, the presence of a cavitary disease, history of previous TB and foreign-born patients or non-foreign patients [3, 4, 5, 8, 10, 11]. In Cantabria, the native population was more likely to be recently infected, in agreement with other reports [3, 11], but unlike other European countries [13, 32]. We compared our MIT clustered patterns with MIT of the SITVIT2 database [29]. This database contains a total of 2379 12-MIRU-VNTR patterns (from 8,161 clinical isolates) from 87 countries of patient origin [29]. We found that the Cantabria population of MTBC share some of its genotypes with other Spanish cities. Viedma *et al*. described Madrid 1 and Madrid 2 spoligopatterns with their corresponding MIT as probable specific clonal complexes in Spain [31]. Lopez Calleja et al. described a strain, *MTZ*, causing a large cluster (85 isolates) in Zaragoza, which is also represented (MIT157) in our population [30]. Another significant finding in our phylogeoraphic structure is that we shared the majority of the clustered patterns with Belgium and Great Britain. One may speculate that the important political-economic relations between Cantabria and these countries in the 16th to 18th centuries could explain this finding [32].

A comparison of our findings with global phylogeography distribution of *M*. *tuberculosis* lineages using the MIRU-VNTR*plus* database indicates that most of the patients (98%) were infected by the Euro-American lineage, which is in agreement with other contemporary studies using MIRU-VNTR in Europe [33, 34] and other continents [35, 36]. Recent literature describes that the East Asian and Euro-American lineages could be more virulent than the Indo-Oceanic and West-African lineages (23) thereby promoting dissemination.

Clonal complexity, understood as mixed infections with more than one strain and simultaneous presence of clonal variants, is widely described in literature [37–39]. MIRU-VNTR has been reported as a rapid, simple and sensitive tool for revealing this matter [17]. In our study, we found a rate of microevolution events of 7.1%, which is similar to countries with a high incidence of TB cases [28, 38]. While these microevolution events could have developed in context of different clinical circumstances [37–39], they are not always limited to specific clinical epidemiological situations [40]. In our study, two cases have previously had TB and two were from countries with high incidence of TB. The remaining two cases were patients with a pulmonary transplant and cavitary pulmonary TB, respectively. Variants could have appeared due to a delay in diagnosis or during the latent phase after infection [19], but we are cautious with these interpretations.

Furthermore, it has been established that a genetic association (polymorphism in CC chemokine ligand 5) plays an important role in pulmonary TB susceptibility in our region, a community with conserved genetic background [41]. Our findings support the idea that predisposition to TB is a complex and multifactorial outcome of interactions by factors such as human genetic determinants [41], bacterial determinants of virulence (predominance of lineage Euro-American) and unknown environmental factors.

The present study has several limitations. First, we used only one method to analyze the clonal structure of the isolates. Second, clonal variants described in this study were found in a single isolate per patient, so the chance that results of MIRU-VNTR had epidemiological and/or clinical repercussions is uncertain. Third, only MTBC strains from culture positive cases could be genotyped, thus in theory, some transmission could occur from not include cases. This is a limitation for all genotyping studies, and we do not believe is will change conclusions significantly. Culturing is for free working against selection bias.

## Conclusion

In summary, our findings suggest that transmission of MTBC is well-controlled in Cantabria. The majority of TB patients were born in Spain and the transmission in this group was higher than in the immigrants group. The population structure of MTBC in Cantabria has a low diversity consisting of principal clonal lineages with the predominance of the Euro American lineage, sharing strain patterns with clonal complexes of Spanish isolates as well as American, European and African isolates.

## Supporting Information

S1 Data MIRU Data. (XLSX)
